# The evolution of cooperation in the unidirectional linear division of labour of finite roles

**DOI:** 10.1098/rsos.220856

**Published:** 2023-03-08

**Authors:** Md Sams Afif Nirjhor, Mayuko Nakamaru

**Affiliations:** School of Environment and Society, Tokyo Institute of Technology, 3-3-6, Shibaura, Minato, Tokyo 108-0023, Japan

**Keywords:** replicator equation of asymmetric games, sanction systems, cooperation in linear network

## Abstract

Evolution of cooperation is a puzzle in evolutionary biology and social sciences. Previous studies assumed that players are equal and have symmetric relationships. In our society, players are in different roles, have an asymmetric relationship and cooperate together. We focused on the linear division of labour in a unidirectional chain that has finite roles, each of which is assigned to one group with cooperators and defectors. A cooperator in an upstream group produces and modifies a product, paying a cost of cooperation, and hands it to a player in a downstream group who obtains the benefit from the product. If players in all roles cooperate, a final product can be completed. However, if a player in a group chooses defection, the division of labour stops, the final product cannot be completed and all players in all roles suffer damage. By using the replicator equations of the asymmetric game, we investigate which sanction system promotes the evolution of cooperation in the division of labour. We find that not the benefit of the product but the cost of cooperation matters to the evolutionary dynamics and that the probability of finding a defector determines which sanction system promotes the evolution of cooperation.

## 1. Introduction

Organisms including humans, plants and bacteria cooperate [1–3]. Especially, human society is built on large-scale cooperation [4,5]. In the modern world, humans can often work with any other human from around the globe, regardless of country, ethnicity, language and genetics. However, the evolution of cooperation in human society has remained somewhat puzzling.

The Prisoner’s Dilemma [6] (hereafter, PD game) can describe why individuals fail to cooperate; if both are cooperators, their payoff is *R*; if both are defectors, their payoff is *P*. The payoff of a defector playing the PD game with a cooperator (*T*) is higher than the cooperator (*S*), and the order of the payoffs is *T* > *R* > *P* > *S*. As a result, players tend to choose defection rather than cooperation regardless of the opponent’s choice. Then, this game has its solution in mutual defection, although mutual cooperation would have given a better payoff for both. In other words, individual rationality is creating collective irrationality. This is called the social dilemma [7]. However, cooperation exists in our society. What mechanism promotes the evolution of cooperation?

There are five possible mechanisms for creating cooperation in social dilemmas naturally, according to Nowak [8] and Rand & Nowak [9]. They are kin selection [10], direct reciprocity (e.g. [11–13]), indirect reciprocity [14–18], network reciprocity [19–25] and group selection (e.g. [26,27]). Apart from these, using sanction to create cooperation has been studied [28–39].

Many previous theoretical studies about the evolution of cooperation considered that two or more players who have the same position as well as having the same payoff matrix regarding those choices. Rather, in reality, players take different positions, which the asymmetric game can describe. For instance, parental investment to care for their offspring was analysed by the asymmetric game by means of evolutionary game theory [40]. It is because the fitness of the male is different from the female in parental care; for example, the female reproduces larger gametes incurring a larger cost, but the male reproduces smaller gametes incurring a smaller cost. This asymmetry is caused by sexual role, depending on the survivorship, the number of eggs and the chance to mate with other females. Another example of the asymmetric relationship among players is cooperation among different social roles where hierarchical social relationships exist [41–43].

The division of labour is one of the other examples of the asymmetric relationship between players [41,44,45]. Because, as different players in different roles obtain different benefits and costs, the asymmetric game can be used for this system. In the division of labour, one player does not complete the whole role alone, but the whole role is divided among many smaller roles and each player is allocated to one role or more, each player only completes his/her role, and all players’ collaboration leads to the completion of the whole role. The division of labour can be observed not only in our human society but also in animals, plants and bacteria [46–48].

In our human society, the historical and ethnographic evidence of division of labour’s presence in many pre-industrial societies and being associated with their development was shown in the previous studies (e.g. [49,50]). In our present world, there are many examples of division of labour, one of which is the division of labour by gender [45]. The industry-centred modern economy has its base in specialization, which is the result of the division of labour. There are intra-industry as well as inter-industry trade and specialization, in the global production networks. Thus, domestic as well as international division of labour is present. The supply chains observed in any industry are an example of the division of labour in the modern economy, where the intra-product specialization can also be seen [51]. It also emerged in societies without centralized institutions such as governments [52]. Here comes the need for cooperation in the division of labour. However, the division of labour is a rather unexplored field in evolutionary game theory, especially when there are three or more roles in a system.

To our knowledge, Nakamaru *et al.* [53] was the first to explore the linear division of labour, where each subtask must be performed in sequence in a line, using the evolutionary game theory. They take the industrial dumping system in Japan as an example. In the industrial dumping system, five subtasks are needed to treat the waste completely. Then, there are companies which are experts in one of five subtasks. A company in subtask 1 asks a company in subtask 2 to treat the waste, paying a monetary cost. The company in subtask 2 accepts the offer and receives the money from the company in subtask 1. After the company finishes treating the waste with paying the cost of treatment, the company asks a company in subtask 3 to treat it, giving money to the company in subtask 3. This process continues. If a company in subtask 4 asked a company in subtask 5 to treat the waste and the company in subtask 5 accepts the offer and completes the task, the industrial waste is treated successfully and completely. If a company in one subtask, for example, dumps the waste illegally, all companies have damage from the illegal dumping. Nakamaru *et al.* [53] considered three subtasks to make the model simple, assuming that a large number of players are in each group where one subtask is assigned to players. A player in the group with subtask 1 chooses a player from the group of subtask 2 and asks the player in subtask 2 to treat the industrial waste properly, giving money to the player. After the player in subtask 2 finishes treating the waste, the player asks a player in subtask 3 to treat the waste properly. If the player in subtask 3 finishes treating it, paying the cost of treatment, the division of labour is completed. Players update their strategy, imitating the strategy of a player with a higher payoff in the group of the same subtask. However, if a player in one of the subtasks does not treat the waste and illegally dumps it, all players in the three subtasks suffer the damage from the illegal dumping and a restoration fee is imposed on all players in all subtasks. As they regard treating the waste properly as cooperation and dumping it illegally as defection, this system can be studied by means of ‘the evolution of cooperation’. As the payoffs of different players in different groups are asymmetric, this system can be modelled by the replicator equations for asymmetric games. They made concrete assumptions to fit the real industrial dumping system, and investigated if either of two existing sanction systems, namely the producer responsibility system and actor responsibility system, can promote the evolution of cooperation by means of the replicator equations for asymmetric games. In the former system if defection happens in the linear chain, whoever defects, the player in the first group gets punished by the supervision. In the later system, if defection happens, the defector is detected and gets punished by the supervision. It was shown that the sanction systems, especially the producer responsibility system when it is almost impossible to monitor and detect defectors, can promote cooperation more than the actor responsibility system. Hereafter, the former sanction system is called the first role sanction system; the latter is the defector sanction system.

In this study, we generalize the three groups model into any countable number of groups which are in line. We assume that players in one group play one role and that a player in an upstream group interacts with a player in a downstream group. We also generalize the model which can be applied to other systems besides the industrial damping system and make a simple assumption. We will investigate whether the sanction systems can promote the evolution of cooperation or not.

There are some evolutionary game theoretical studies that seem similar to our framework. The effect of network structures on creating cooperation in asymmetric social interactions has been studied (e.g. [25,54]). Their studies seem to include ours; however, it is not true. In their studies, each player is in each vertex, a player imitates the strategy of the neighbour who is a partner in the game if the payoff is higher than that of the player. For example, Su *et al.* [25] concentrated on how edge orientation, directionality, regularity and properties of graphs will change the evolution of cooperation, assuming that the cooperation cost and benefit for all players are the same. While in our study, each group is in the line and has a large number of players. A player imitates the strategy of others in the group. A player chosen randomly from an upstream group never plays the game with another player in the same group but does with a player chosen randomly in the downstream group. The players in different roles have different cooperation costs and benefits. Therefore, from the viewpoint of mathematical modelling, our study proposes a model which the previous studies about the evolution of cooperation in the network structure have not assumed.

We have only compared our study with the previous ones which assumed that each player is located at each node of the network. There are studies about the evolution of cooperation assuming that each group is located at each node of the two-dimensional lattice, and players move to their neighbouring groups (e.g. [55]). These studies did not assume a player in a group plays the game with another in a different group.

In our model, players play the game sequentially, but our game is different from the sequential game studied previously in economics where the information of the previous player is available to the later player (e.g. [56,57]). In ‘the two-person sequential game’, for example, a player chooses the strategy after the opponent chooses the strategy, and upon that information changes his or her strategy [56,57]. After two players choose their strategy, they can receive the payoff.

Therefore, our study gives a new perspective that the division of labour can be studied from the viewpoint of ‘the evolution of cooperation’. Moreover, we can propose a model which the previous studies had not assumed and analysed from the viewpoint of the mathematical models in ‘the evolution of cooperation’.

## 2. Three models

### 2.1. Baseline system

**Figure 1.**
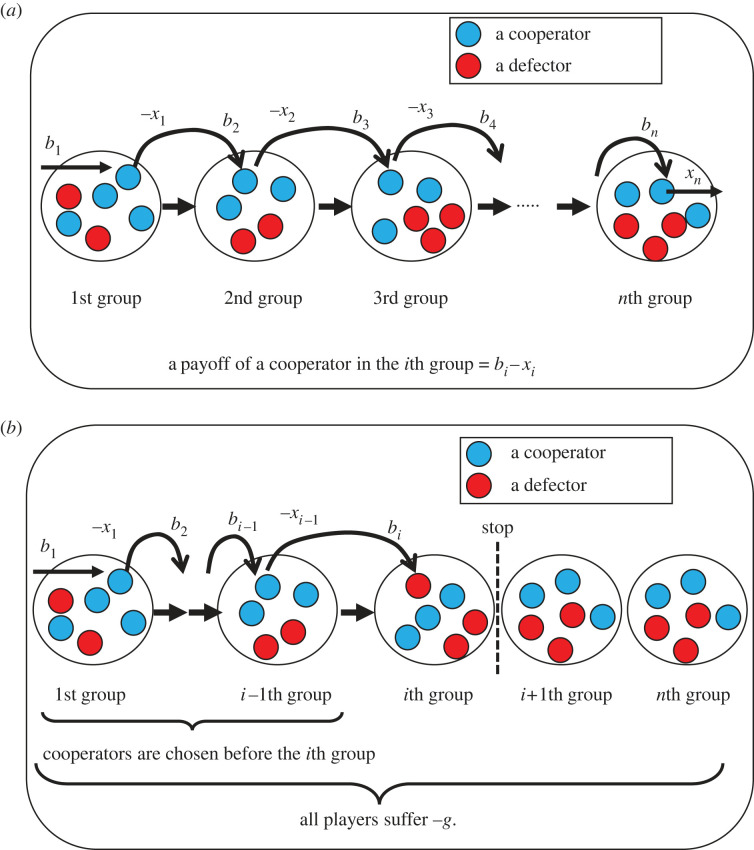
(*a*) Linear graph showing *n* division of labour when cooperators are chosen in all groups. The payoff of a cooperator in the *i*th group is *x*_*i*−1_ − *x*_*i*_. (*b*) Linear graph showing *n* division of labour when cooperators are chosen before the *i*th group and then a defector is chosen in the *i*th group. Once a defector is chosen, the whole system is broken. As a result, all players suffer −*g*.

We present the baseline system (see figure 1), where there are *n* roles (n∈N and n≥2). Here **N** is the set of natural numbers. For *n* = 1, there is no linear division of labour. There are *n* groups in the whole population. Each group is allocated to one role, and the group size is infinite. Each group consists of cooperators and defectors. We define cooperators as players who only cooperate, and defectors as players who only defect. We do not assume a mixed strategy in which players choose either cooperation or defection by probability. The frequency of cooperators in group *i* is *i*_*c*_ and the frequency of defectors is *i*_*d*_. Here, *i*_*c*_ + *i*_*d*_ = 1. It is assumed that one player chosen randomly from the group *i* interacts with a player chosen randomly from the group *i* + 1 (1 ≤ *i* < *n*).

We consider that the final product or service is produced through the division of labour; if players in all roles cooperate together to produce the product or service, the final product or service can be completed (figure 1*a*). A cooperator in an upstream group produces and modifies the product or service, paying a cost of cooperation, and gives it to a player in a downstream group. Let *x*_*i*_ be defined as a cost of cooperation by a cooperator in the group *i*. The value of the product or service is regarded as the benefit to the player in the group *i* + 1, *b*_*i*+1_. In this case, the net benefit of cooperators in the group *i* is *b*_*i*_ − *x*_*i*_ (table 1).

**Table 1.** The payoff matrix in the baseline system for a player in the *i*th group.

cases	all being cooperator except the *i*th	a defector before *i*th	a defector after *i*th
*i*th player cooperator	*b*_*i*_ − *x*_*i*_	−*g*	*b*_*i*_ − *x*_*i*_ − *g*
*i*th player defector	*b*_*i*_ − *g*	−*g*	*b*_*i*_ − *g*

For the player in the first group, the benefit comes from the source. The net benefit of the cooperator in group 1 is *b*_1_ − *x*_1_. In the *n*th group, a cooperator pays a cost, *x*_*n*_, to produce the final product, and the payoff of the cooperator is *b*_*n*_ − *x*_*n*_, where the player sells the final product and gets benefit *b*_*n*_.

If a defector is chosen randomly from group *i*, the defector receives the benefit *b*_*i*_ and s/he does not cooperate with a player in group *i* + 1 (figure 1*b*). As a result, the division of labour fails and the whole system is broken down. As a result, all players in all the groups get the same negative effect *g* in their payoff; *g* can be interpreted as a bad reputation because the incomplete work gives a bad reputation to all members in the division of labour. The payoff of the defector is *b*_*i*_ − *g* (table 1). After the defector is chosen from group *i*, the players in the later groups will not choose either cooperation or defection, and there can only be one acting defector player in the chain of the linear division of labour here. Therefore, the net payoff of players after defection occurs is −*g* (table 1).

Table 1 indicates that, when *g* < *x*_*i*_, the game can be the PD game. Otherwise, mutual cooperation is always the best. In other words, when *g* > *x*_*i*_ for all *i*s, the cooperators’ payoff is higher than the defectors. Therefore, we assume that *g* < *x*_*i*_, which means that the baseline system has a dilemma situation.

For calculating the payoff matrix of a player in group *i* we need to consider three cases. First, the probability that all of the players in the rest of the groups are cooperators in the chain of the division of labour is defined as *c*_*i*_, which is the product of the frequencies of the cooperators of the groups except *i*. Therefore, $c_{i}=\Pi_{\;j=1}^{i-1}j_{c}\Pi_{\;j=i+1}^{n}j_{c}$. Second, there are defectors in the groups before the group *i*, with probability *d*_*ib*_. Thus, it is $d_{ib}=1-c_{ib}=1-\Pi_{\;j=1}^{i-1}j_{c}$, where *c*_*ib*_ is the probability that all the players chosen from the previous groups are cooperators. Third, the probability that there are defectors in the groups after the group *i* is *d*_*ia*_, where no defectors are in the previous groups. Thus, $d_{ia}=c_{ib}(1-c_{ia})=\Pi_{\;j=1}^{i-1}j_{c}(1-\Pi_{\;j=i+1}^{n}j_{c})$, where *c*_*ia*_ is the probability of not having defectors after *i*. Here, *c*_*i*_ + *d*_*ib*_ + *d*_*ia*_ = 1.

After each player randomly chosen from each group interacts with a player randomly chosen from the next downstream group, the expected payoff of each player in each group can be calculated. Then, within each group, players decided to imitate a strategy of others, proportional to the expected payoff relative to the total payoff in the group. Here the random change of the strategy or mutation does not occur. Therefore, this interaction can be described by the replicator dynamics of the asymmetric game without mutation [58].

The replicator equation of a cooperator in the group 1 in the baseline system is as follows:

2.1 $$\frac{d1_{c}}{dt}=1_{c}(1-1_{c})(P_{1_{c}}-P_{1_{d}})=1_{c}(1-1_{c})\{c_{1}g-x_{1}\},$$

where the average payoff of cooperator in the group 1, $P_{1_{c}}=c_{1}(b_{1}-x_{1})+d_{1a}(b_{1}-x_{1}-g)$ and the average payoff of the defector in the group 1, $P_{1_{d}}=b_{1}-g$.

The replicator equation for cooperators in the group *i* is (when 1 < *i* < *n*) as follows:

2.2 $$\frac{di_{c}}{dt}=i_{c}(1-i_{c})(P_{i_{c}}-P_{i_{d}})=i_{c}(1-i_{c})\{c_{i}g-x_{i}(1-d_{ib})\},$$

where the average payoff of the cooperators in the group *i* is $P_{i_{c}}=c_{i}(b_{i}-x_{i})+d_{ib}(-g)+d_{ia}(b_{i}-x_{i}-g)$ and the average payoff of the defectors in the group *i* is $P_{i_{d}}=c_{i}(b_{i}-x_{i}-g)+d_{ib}(-g)+$ $d_{ia}(b_{i}-x_{i}-g)$.

The replicator equation for the cooperators in the group *n* is as follows:

2.3 $$\frac{dn_{c}}{dt}=n_{c}(1-n_{c})(P_{n_{c}}-P_{n_{d}})=n_{c}(1-n_{c})\{c_{n}(g-x_{n})\},$$

where the average payoff of the cooperators in the group *n*, $P_{n_{c}}=c_{n}(b_{n}-x_{n})+d_{nb}(-g)$ and the average payoff of the defectors in the group *n*, $P_{n_{d}}=c_{n}(b_{n}-x_{n}-g)+d_{nb}(-g)$.

### 2.2. The defector sanction system

Next we focus on two sanction systems, namely the defector sanction system and the first role sanction system. In the defector sanction system, the defector in the chain of the linear division of labour gets punished with a fine *f*, where (*f* > 0) and the finding probability of the defector is *ρ*. In some types of linear division of labour, where monitoring a defector is too hard, *ρ* is very low compared with other parameters.

For the defector sanction system, the payoffs are given in a similar way as the baseline except the sanction; adding the sanction of *ρf* to the defector’s payoff. In the defector sanction system, the payoff matrix for a player in group *i* is in table 2.

**Table 2.** The payoff matrix in the defector sanction system for a player in the *i*th group.

cases	all being cooperator except the *i*th	a defector before *i*th	a defector after *i*th
*i*th player cooperator	*b*_*i*_ − *x*_*i*_	−*g*	*b*_*i*_ − *x*_*i*_ − *g*
*i*th player defector	*b*_*i*_ − *g* − *ρf*	−*g*	*b*_*i*_ − *g* − *ρf*

The replicator equation for the cooperators in the group 1 is as follows:

2.4 $$\frac{d1_{c}}{dt}=1_{c}(1-1_{c})(P_{1_{c}}-P_{1_{d}})=1_{c}(1-1_{c})\{c_{1}g+\rho f-x_{1}\},$$

where the average payoff of the cooperators in the group 1, $P_{1_{c}}=b_{1}-x_{1}-d_{1a}g$ and the average payoff of the defectors in the group 1, $P_{1_{d}}=b_{1}-g-\rho f$.

In the defector sanction system, the replicator equation for the cooperators in the group *i* is (when 1 < *i* < *n*) as follows:

2.5 $$\frac{di_{c}}{dt}=i_{c}(1-i_{c})(P_{i_{c}}-P_{i_{d}})=i_{c}(1-i_{c})\{c_{i}g+(\rho f-x_{i})(1-d_{ib})\},$$

where the average payoff of cooperators in the group *i* is $P_{i_{c}}=c_{i}(b_{i}-x_{i})+d_{ib}(-g)+d_{ia}(b_{i}-x_{i}-g)$ and the average payoff of the defectors in the group *i* is $P_{i_{d}}=c_{i}(b_{i}-g-\rho f)+d_{ib}(-g)+d_{ia}(b_{i}-g-\rho f)$.

In the defector sanction system, the replicator equation for the cooperators in the group *n* is as follows:

2.6 $$\frac{dn_{c}}{dt}=n_{c}(1-n_{c})(P_{n_{c}}-P_{n_{d}})=n_{c}(1-n_{c})\{c_{n}(g+\rho f-x_{n})\},$$

where the average payoff of the cooperators in the group *n*, $P_{n_{c}}=c_{n}(b_{n}-x_{n})+d_{nb}(-g)$ and the average payoff of the defectors in the group *n*, $P_{n_{d}}=c_{n}(b_{n}-g-\rho f)+d_{nb}(-g)$.

### 2.3. The first role sanction system

In the first role sanction system, the fine is the same as the defector sanction system, *f*, and a defection in the chain of the linear division of labour is always found with probability 1, because the final product or service does not appear if defection occurs, and players can know that defection occurs without monitoring a defector. Therefore, the finding probability is one. The player in the first role always gets punished for the defection, no matter which role defected. For example, this sanction system is executed to prevent illegal dumping in Japan [53].

For the first role sanction system for the generalized *i*th player, the payoff matrix is the same as the baseline except that for group 1. If anyone defects, the first role gets punished, and sanction *f* appears in the first group’s payoff matrix (table 3). The payoff matrix for a player in the group 1 in the first role sanction system is in table 3, and for a player in the group *i* (2 ≤ *i* ≤ *n*) is in table 4.

**Table 3.** The payoff matrix in the first role sanction system for group 1.

cases	all being cooperator except the first	a defector after the first
first player cooperator	*b*_1_ − *x*_1_	*b*_1_ − *x*_1_ − *g* − *f*
first player defector	*b*_1_ − *g* − *f*	*b*_1_ − *g* − *f*

**Table 4.** The payoff matrix in the first role sanction system for a player in the group 2 ≤ *i* ≤ *n*.

cases	all being cooperator except the *i*th	a defector before *i*th	a defector after *i*th
*i*th player cooperator	*b*_*i*_ − *x*_*i*_	−*g*	*b*_*i*_ − *x*_*i*_ − *g*
*i*th player defector	*b*_*i*_ − *g*	−*g*	*b*_*i*_ − *g*

The replicator equation for a cooperator in the group 1 is as follows:

2.7 $$\frac{d1_{c}}{dt}=1_{c}(1-1_{c})(P_{1_{c}}-P_{1-d})=1_{c}(1-1_{c})\{c_{1}(g+f)-x_{1}\},$$

where the average payoff of the cooperators in the group 1, $P_{1_{c}}=b_{1}-x_{1}+d_{1a}(-f-g)$ and the average payoff of the defectors in the group 1, $P_{1_{d}}=b_{1}-g-f$.

The replicator equation for the cooperators in the groups 1 < *i* < *n* and group *n* are the same as the baseline model. The replicator equation for the cooperators in the group *i* is (when 1 < *i* < *n*) as follows:

2.8 $$\frac{di_{c}}{dt}=i_{c}(1-i_{c})(P_{i_{c}}-P_{i_{d}})=i_{c}(1-i_{c})\{c_{i}g-x_{i}(1-d_{ib})\},$$

where the average payoff of the cooperators in the group *i* is $P_{i_{c}}=c_{i}(b_{i}-x_{i})+d_{ib}(-g)+d_{ia}(b_{i}-x_{i}-g)$ and the average payoff of the defectors in the group *i* is $P_{i_{d}}=c_{i}(b_{i}-x_{i}-g)+d_{ib}(-g)+d_{ia}(b_{i}-x_{i}-g)$.

The replicator equation for the cooperators in the group *n* is as follows:

2.9 $$\frac{dn_{c}}{dt}=n_{c}(1-n_{c})(P_{n_{c}}-P_{n_{d}})=n_{c}(1-n_{c})\{c_{n}(g-x_{n})\},$$

where the average payoff of the cooperators in the group *n*, $P_{n_{c}}=c_{n}(b_{n}-x_{n})+d_{nb}(-g)$ and the average payoff of the defectors in the group *n*, $P_{n_{d}}=c_{n}(b_{n}-x_{n}-g)+d_{nb}(-g)$.

Table 5 shows the parameter list in our model.

**Table 5.** Parameters.

*n*	the number of groups, (n∈N and n≥2, here N is the set of natural numbers)
*i*	index of groups, 1 ≤ *i* ≤ *n*
*b*_*i*_	the benefit given by the *i*th group player to the (*i* + 1)th group player
*x*_*i*_	the cost of cooperation for the *i*th group player
*i*_*c*_	the frequency of cooperators in the *i*th group
*i*_*d*_	the frequency of defectors in the *i*th group
*g*	a damage for all players once one defector appears in the line
*f*	the amount of punishment
*ρ*	the probability of catching a defector
*c*_*i*_	the probability of having all cooperators in the line except the *i*th group
*d*_*ib*_	the probability of having a defector in the line before the *i*th group
*d*_*ia*_	the probability of having a defector in the line after the *i*th group

## 3. Results

### 3.1. The summary of results in all three systems

We find four sorts of equilibrium in all three systems in *n* ≥ 3 (see appendix A). One is the all cooperation equilibrium which we represent as [1_*c*_, 2_*c*_, …, *n*_*c*_] = [1, 1, …, 1]. The second one is the first group defection equilibirum which we represent as [0, ∗, …, ∗] where ‘∗’ is any value between 0 and 1. In equilibrium, *i*_*c*_ is neutral (*i* ≥ 2) because the game stops after the player in the group 1 chooses defection and the players in the later roles gets the same payoff regardless of the behaviour. As a result, *i*_*c*_ neutrally converges to any value between 0 and 1 (*i* > 1). The third one is the cooperation–defection mixed equilibrium which is represented as [1_*c*_, 2_*c*_, …, (*j* − 1)_*c*_, *j*_*c*_, (*j* + 1)_*c*_, …, *n*_*c*_] = [1, 1, …, 1, 0, ∗, · · · , ∗], where *j* is between 2 and *n* − 1. The fourth is [1_*c*_, 2_*c*_, …, (*n* − 1)_*c*_, *n*_*c*_] = [1, 1, …, 1, 0] which is named the last group defection equilibrium. Appendix A proves that this system only has four types of equilibrium points. We analyse the local stability of these four equilibria with the Jacobian matrix (see appendix B). Table 6 presents the summary of the analyses.

**Table 6.** Local stability conditions for the general model.

equilibrium	baseline	defector sanction	first role sanction
[0, ∗, …, ∗]	always stable	*ρf* < *x*_1_ − *c*_1_*g*	*c*_1_(*g* + *f*) < *x*_1_
[1, …, 1, 0, ∗, …, ∗]	always unstable	$\max{\left\{x_{i}\right\}}_{i=1}^{\;j-1}<\rho f<x_{j}-c_{j}g$	always unstable
[1, …, 1, 0]	always unstable	$\max{\left\{x_{i}\right\}}_{i=1}^{n-1}<\rho f$	always unstable
		$\&\;x_{n}>g+\rho f$	
[1, …, 1]	$g>\max{\left\{x_{i}\right\}}_{i=1}^{n}$	$\rho f+g>\max{\left\{x_{i}\right\}}_{i=1}^{n}$	*f* + *g* > *x*_1_ & $g>\max{\left\{x_{i}\right\}}_{i=2}^{n}$

*j* is the first defector in [1, …, 1, 0, ∗, …, ∗].

Table 6 shows that the first group defection equilibrium is one stable equilibrium in the baseline. It is also stable in the first role sanction system when *c*_1_(*g* + *f*) < *x*_1_. However, it is stable in the defector sanction system, if *ρf* < *x*_1_ − *c*_1_*g*.

The cooperation–defection mixed equilibrium is unstable in both the baseline and the first role sanction system. It is stable in the defector sanction system when *ρf* < *x*_*j*_ - *c_j_g* and $\max{\left\{x_{i}\right\}}_{i=1}^{\;j-1}<\rho f$, where a player in the group *j* defects and everyone cooperates in the groups before the group *j*.

The last group defection equilibrium is unstable in the baseline and the first role sanction system. It is stable in the defector sanction system when $\max{\left\{x_{i}\right\}}_{i=1}^{n-1}<\rho f$ and *x*_*n*_ > *g* + *ρf*.

The all cooperation equilibrium is locally stable if $g>\max{\left\{x_{i}\right\}}_{i=1}^{n}$ in the baseline system. This means that players in all groups are changed to cooperators in equilibrium when *g*, the loss caused by defectors, is higher than the cost of cooperator in the all cooperation equilibrium. However, we consider the PD situation in the baseline system, and then we can assume that *g* < *x*_*i*_. This indicates that all cooperation equilibrium is unstable in the baseline. It is stable in the defector sanction system when $\rho f+g>\max{\left\{x_{i}\right\}}_{i=1}^{n}$, which is held even though *g* < *x*_*i*_. Therefore, if *ρf* is large enough, the defector sanction system can promote the evolution of cooperation.

The all cooperation equilibrium is stable in the first role sanction system when *f* + *g* > *x*_1_ and $g>\max{\left\{x_{i}\right\}}_{i=2}^{n}$. This indicates that the first role sanction system promotes the evolution of cooperation more than the baseline system. If we consider the assumption, *g* < *x*_*i*_, which satisfies the condition of the PD game, the all cooperation equilibrium is considered stable if the condition that *x*_1_ − *f* < *g* < *x*_1_ is possible in the first role sanction system.

Appendix B and table 6 suggest that the benefit given by the *i*th group player to the (*i* + 1)th group player, *b*_*i*_, is cancelled out and does not influence the local stability of each equilibrium point.

To understand the dynamics well, we will discuss three special cases as well as other cases about the cost of cooperation in the following sections.

### 3.2. Three special cases

#### 3.2.1. The cost of cooperation is the same for all the groups

The simplest assumption is that *x*_*i*_ is the same for all the 1 ≤ *i* ≤ *n*; *x*_1_ = · · · = *x*_*n*_ = *x*. After exploring the local stability conditions for each equilibrium in each of the three systems, we can summarize the results as table 7, which represents that the first group defection equilibrium is a stable equilibrium in the baseline, and is also stable in the first role sanction system when *c*_1_(*g* + *f*) < *x*. In the defector sanction system, it is locally stable when *ρf* < *x* − *c*_1_*g*.

**Table 7.** Local stability conditions when the cooperation cost is the same for all the groups.

equilibrium	baseline	defector sanction	first role sanction
[0, ∗…, ∗]	always stable	*ρf* < *x − c*_1_*g*	*c*_1_(*g* + *f*) < *x*
[1, …, 1, 0, ∗, …, ∗]	always unstable	always unstable	always unstable
[1, …, 1, 0]	always unstable	always unstable	always unstable
[1, …, 1]	*g* > *x*	*ρf* + *g* > *x*	*g* > *x*

The cooperation–defection mixed equilibrium and the last group defection equilibrium are unstable in all the systems. This indicates that once cooperation in group 1 starts, the all cooperation equilibrium will be stable. It is intuitive because all players face the same cost −*g* once a player chooses defection and the cost of cooperation *x* is the same in all the groups. Therefore, the players in the later groups will follow the cooperators in the first group. It is meaningful to punish a defector in the earliest group to promote cooperation. Therefore, the first role sanction system works.

Our analysis suggests that the all cooperation equilibrium is stable in the baseline system and in the first role sanction system only when *g* > *x*. As we assume that *g* < *x*, which meets the PD game, the equilibrium is not stable. It is stable when *ρf* + *g* > *x* for the defector sanction system.

#### 3.2.2. The cost of cooperation is lower in higher *i*

Here, we consider the special case where the cost in a downstream group decreases in the linear division of labour, *x*_1_ > *x*_2_ > · · · > *x*_*n*_.

After exploring the local stability conditions for each equilibrium in each of the three systems, we can summarize the results as table 8, which represents that the first group defection equilibrium is a stable equilibrium in the baseline, and is also stable in the first role sanction system when *c*_1_(*g* + *f*) < *x*_1_. In the defector sanction system, it is locally stable when *ρf* < *x*_1_ − *c*_1_*g*.

**Table 8.** Local stability conditions when the cooperation cost decreases in the downstream.

equilibrium	baseline	defector sanction	first role sanction
[0, ∗, …, ∗]	always stable	*ρf* < *x*_1_ − *c*_1_*g*	*c*_1_(*g* + *f*) < *x*_1_
[1, …, 1, 0, ∗, …, ∗]	always unstable	always unstable	always unstable
[1, …, 1, 0]	always unstable	always unstable	always unstable
[1, …, 1]	*g* > *x*_1_	*ρf* + *g* > *x*_1_	*f* + *g* > *x*_1_ & *g* > *x*_2_

The cooperation–defection mixed equilibrium and the last group defection equilibrium are unstable in all the system.

**Figure 2.**
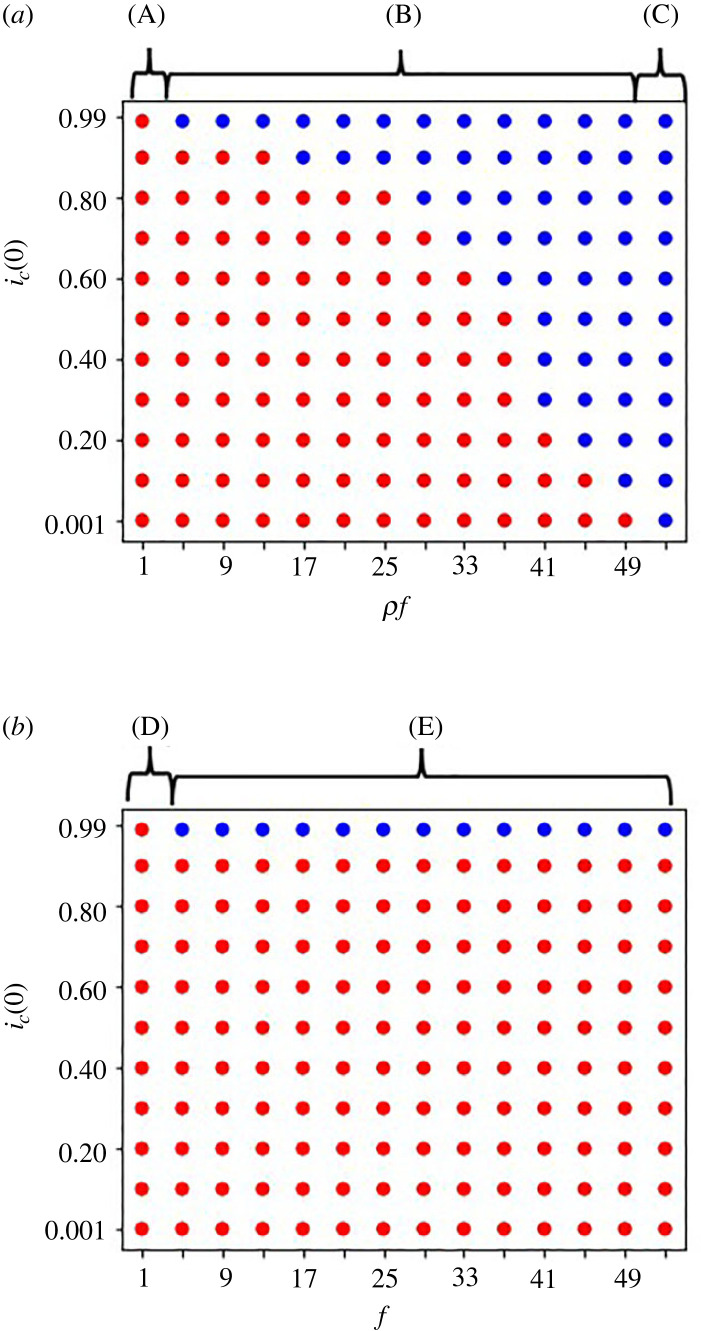
Initial frequency dependency in (*a*) the defector sanction system and (*b*) the first role sanction system with *n* = 10 when the cooperation cost decreases downstream. The horizontal axis is for *ρf* in (*a*) and for *f* in (*b*). The vertical is for the initial frequency of cooperators in group *i*, *i*_*c*_(0), when *i* is an integer between 1 and 10. Blue dots shows when the dynamics evolves into all cooperation and red dots shows when the dynamics evolves into the first group defection equilibrium. The parameters are: *g* = 48, *x*_1_ = 50, *ρ* = 0.001 and *x*_*i*−1_ − *x*_*i*_ = 5 for all *i*s. (A) in (*a*) means *ρf* < *x*_1_ − *g* = 2; (B), *x*_1_ − *g* = 2 < *ρf* < *x*_1_ − *c*_1_*g* = 50; (C), *x*_1_ = 50 < *ρf*. (D) in (*b*) presents *f* < *x*_1_ − *g* = 2; (E), *x*_1_ − *g* = 2 < *f* and *g* > *x*_2_.

Our analysis suggests that the all cooperation equilibrium is stable in the baseline system only when *g* > *x*_1_ because *x*_1_ > *x*_2_ > · · · > *x*_*n*_. When the condition of the PD game is applied, the equilibrium is not stable. It is stable when *ρf* + *g* > *x*_1_ for the defector sanction systems (figure 2*a*), and is stable when *f* + *g* > *x*_1_ and *g* > *x*_2_ for the first role sanction system (figure 2*b*). Figure 2*a*,*b* shows that the same sanction *f* as the first role sanction system cannot create the evolution of cooperation in the defector sanction system, because of the low finding probability of the defector.

By comparison between tables 7 and 8, it is shown that the outcomes of this case (table 8) is the same as table 7 which is the result when the cooperation cost is the same for all the group. The numerical analyses indicate that the parameter *c*_1_ becomes almost zero so that this parameter does not influence the simulation outcomes in the first group defection equilibrium (figure 2).

**Figure 3.**
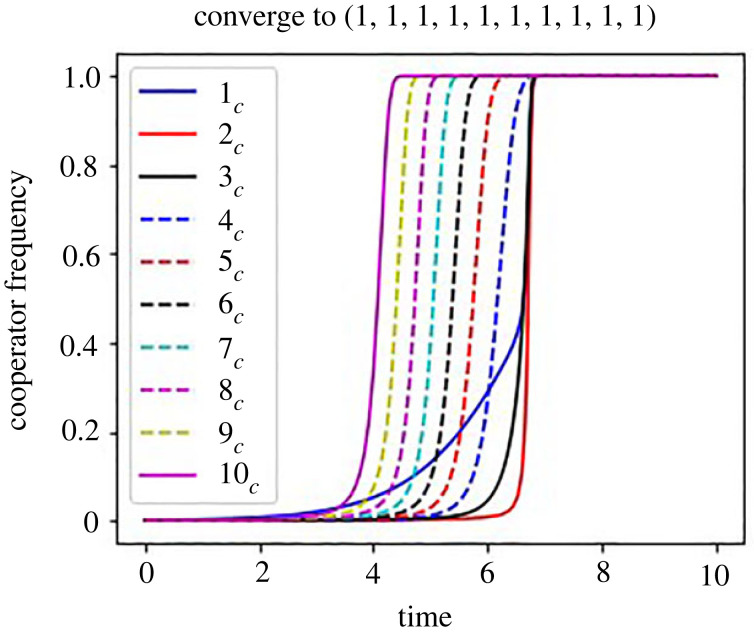
Dynamics of the system with *n* = 10 when cooperation cost decreases downstream in the defector sanction system with *ρf* = 51. Even when the initial frequency of cooperators are rare, the evolution of cooperation happens if *ρf* > *x*_1_. The parameters are: *i*_*c*_(0) = 0.001, *g* = 48, *ρ* = 0.001, *x*_1_ = 50 and *x*_*i*−1_ − *x*_*i*_ = 5 (for all *i*s).

Surprisingly enough, figures 2*a* and 3 show the defector sanction system promotes cooperation even though cooperators are rare in the beginning in *ρf* > *x*_1_. In the region where *ρf* + *g* > *x*_1_ and *ρf* < *x*_1_, the system is bistable, where the sanction needs to be higher to create all cooperation with lower *i*_*c*_(0), and even low sanction can create all cooperation with higher *i*_*c*_(0). Figure 2*a* also shows that the dynamics is independent of the initial condition and goes to all defection in the region of *ρf* + *g* < *x*_1_. Therefore, if the probability of finding and catching a defector is too low and *ρf* is very low, the defector sanction system never promotes cooperation. If the sanction, *ρf*, is large enough to be effective, the defector sanction system promotes cooperation even though the initial frequency of cooperators is low.

Figure 2*b* shows clearly that the first role sanction system creates cooperation only when the initial frequency of cooperators in all the groups is very high. When the *i*_*c*_(0) comes near 0.95, cooperation only evolves with very high sanction *f*. When *i*_*c*_(0) is low, the system goes to first group defection.

#### 3.2.3. The cost of cooperation is higher in higher *i*

Now we consider the model when the cost of cooperation rises in the downstream of the linear chain. Here we assume that, *x*_*i*_ > *x*_*i*−1_ for all *i*s. The local stability conditions for each of the four equilibria are shown in table 9.

**Table 9.** Local stability conditions when the cooperation cost increases in downstream.

equilibrium	baseline	defector sanction	first role sanction
[0, ∗, …, ∗]	always stable	*ρf* < *x*_1_ − *c*_1_*g*	*c*_1_(*g* + *f*) < *x*_1_
[1, …, 1, 0, ∗, …, ∗]	always unstable	*x*_*j*−1_ < *ρf* < *x*_*j*_ − *c_j_g*	always unstable
[1, …, 1, 0]	always unstable	*x*_*n*−1_ < *ρf*	always unstable
		$\&\;x_{n}>g+\rho f$	
[1, …, 1]	*g* > *x*_*n*_	*ρf* + *g* > *x*_*n*_	*g* > *x*_*n*_

*j* is the first defector in [1, …, 1, 0, ∗, …, ∗].

The main difference between the condition where the cost of cooperation increases in downstream groups and the condition where the cost of cooperation decreases in downstream groups is that the defector sanction system makes the cooperation–defection mixed equilibrium as well as the last group defection equilibrium locally stable when the cost of cooperation increases in downstream groups (table 9).

**Figure 4.**
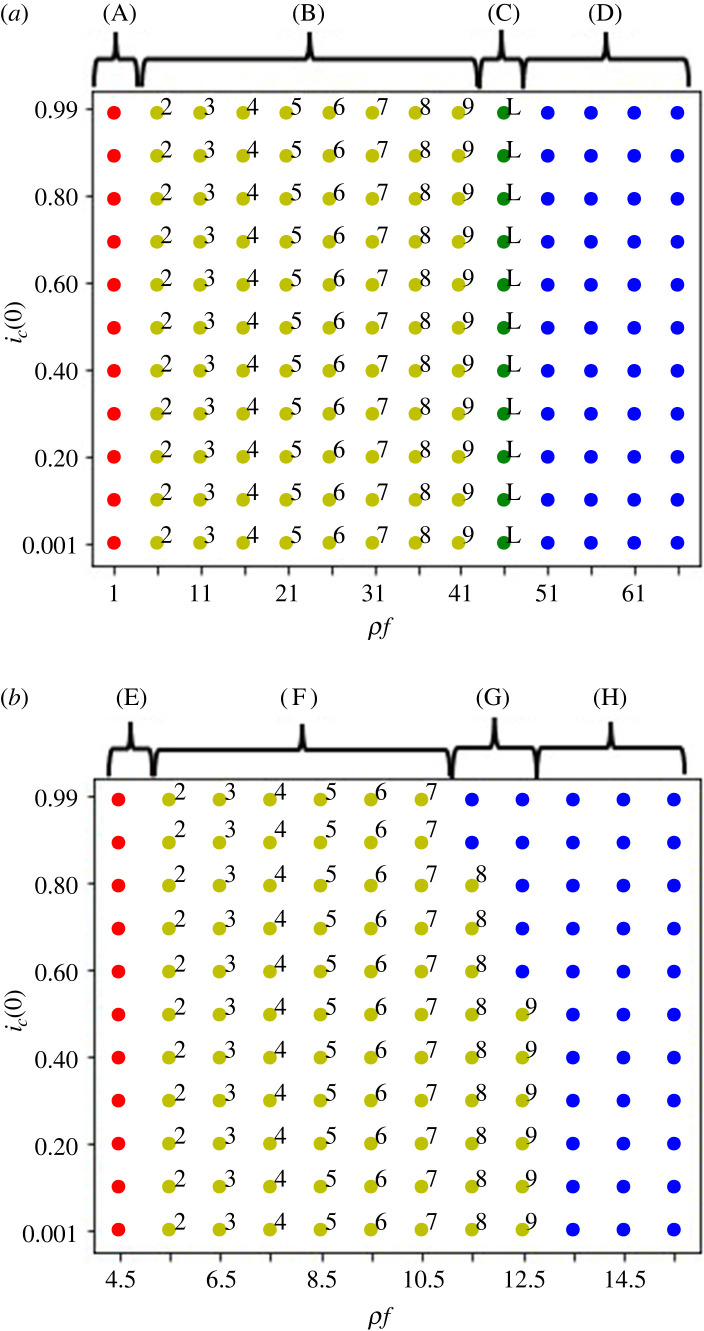

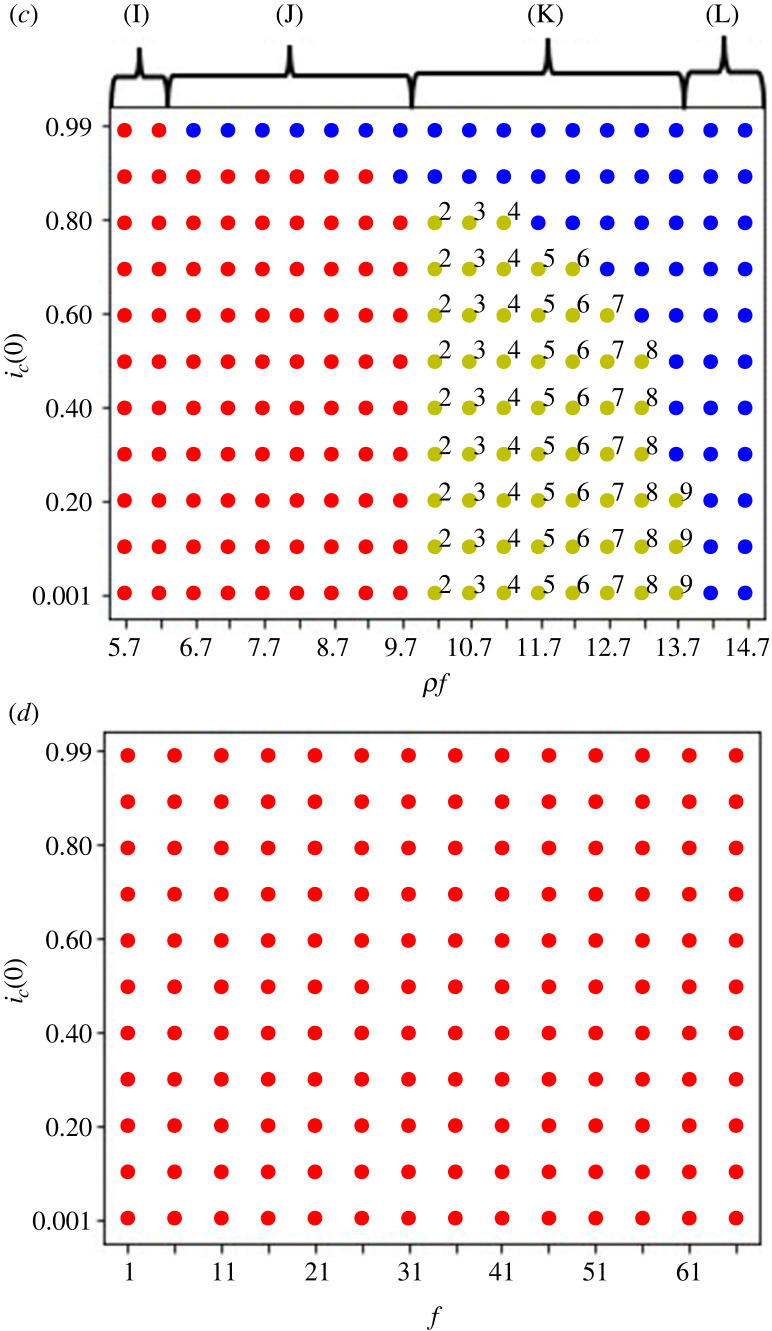
Initial frequency dependency in the defector sanction system in (*a*), (*b*) and (*c*), and in (*d*) the first role sanction system with *n* = 10 when the cooperation cost increases downstream. The horizontal axis is for *ρf* in (*a*), (*b*) and (*c*), and for *f* in (*d*). The vertical axis is for *i*_*c*_(0) when *i* ∈ {1, 2, 3, 4, 5, 6, 7, 8, 9, 10}. The parameters are: *g* = 3, *x*_1_ = 5 and *ρ* = 0.001 for (*a*), (*b*) and (*d*). In (*a*) and (*d*) *x*_*i*_ − *x*_*i*−1_ = 5 for all *i*s. In (*b*) *x*_*i*_ − *x*_*i*−1_ = 1 for all *i*s. In (*c*) *g* = 8, *x*_1_ = 10, *ρ* = 0.001, and *x*_*i*_ − *x*_*i*−1_ = 0.5. Blue dots show when the dynamics evolves into all cooperation and red dots shows when the dynamics evolves into the first group defection equilibrium. The yellow dots show when the dynamics evolves into a cooperation–defection mixed equilibrium. The number (*j*) outside each yellow dot shows all players in the group *j* are first defectors in the cooperation–defection mixed equilibrium. As *x*_*j*−1_ < *ρf* < *x*_*j*_ is the condition for the cooperation–defection mixed equilibrium to be stable where *j* is the first defector. The green dots with the letter ‘L’ show when the dynamics evolves into the last group defection equilibrium. (A) in (*a*) means *ρf* < *x*_1_ = 5; (B), *x*_*j* − 1_ < *ρf* < *x*_*j*_ − *c_j_g*; (C), *x*_*n*-1_< *ρf* < *x_n_* − *g*; (D), *x*_*n*_ − *g* = 47 < *ρf*. (E) in (*b*) means *ρf* < *x*_1_ = 5; (F), *x*_*j* − 1_ < *ρf* < *x*_j_ − *c_j_g*; (G), *x_n_* − *g* < *ρf* < *x*_*n −* 1_ − *c_n_*_− 1_*g*; (H), *x*_*n*_ − *g* < *x_n_*_− 1_ − *c_n_*_− 1_*g* < *ρf.* (I) in (*c*) means *ρf* < *x*_*n*_ − *g* < *x*_1_ − *c*_1_*g*; (J), *x*_*n*_ − *g* < *ρf* < *x*_1_ − *c*_1_*g*; (K), *x*_*n*_ − *g* < *x*_*j* − 1_ < *ρf* < *x_j_* − *c_j_g*; (L), *x_n_* − *g* < *x*_*n* − 1_ − *c*_*n* − 1_*g* < *ρf*.

**Figure 5.**
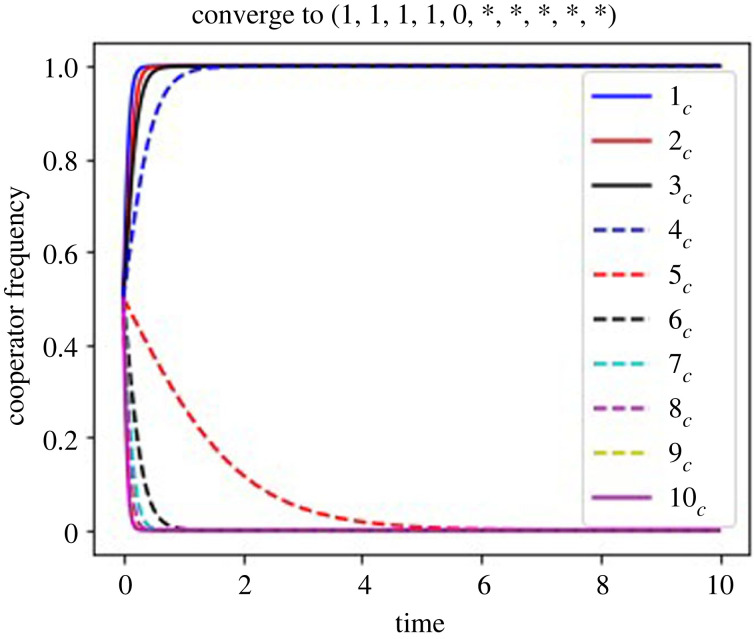
Time evolution in the defector sanction system with *n* = 10 when cooperation cost increases downstream. The horizontal axis is for time and the vertical one is for the frequency of cooperators in each group. *ρf* = 24, *i*_*c*_(0) = 0.5 shows the dynamics evolving into the cooperation–defection mixed equilibrium where all players are cooperators until the group 4 and all are defectors in the group 5 as *x*_4_ < *ρf* < *x*_5_. The parameters are: *g* = 3, *x*_1_ = 5, *ρ* = 0.001 and *x*_*i*_ − *x*_*i*−1_ = 5 for all *i*s.

Figure 4*a* shows that the dynamics in the defector sanction system where all four of the equilibria are present when *x*_*n*−1_ < *x*_*n*_ − *g*; in the region of *ρf* < *x*_1_ the dynamics goes to the first group defection equilibrium, even with very high initial frequency of cooperators in all groups. When *x*_*j*−1_ < *ρf* < *x*_*j*_, and *j* is neither one nor the terminal, all players cooperate till the group *j* − 1 before the group *j* choosing full defection; the cooperation–defection mixed equilibrium is locally stable, as shown in figure 5. When *x*_*n*−1_ < *ρf* and *x*_*n*_ > *g* + *ρf*, the last group defection equilibrium is locally stable. When *ρf* + *g* > *x*_*n*_, all players in all groups go to cooperation even though their initial frequencies of cooperators are low.

When *x*_*n*−1_ > *x*_*n*_ − *g* and *x*_*n*_ − *g* > *x*_1_, figure 4*b* shows the bistable region where both the cooperation–defection mixed equilibrium and the all cooperation equilibrium are locally stable when *x*_*n*_ − *g* < *ρf* < *x*_*j*_ < *x*_*n*−1_, where *j* is the first defector (1 < *j* < *n* − 1). However, the last group defection equilibrium is not locally stable, because the condition for *x*_*n*−1_ < *ρf* and *x*_*n*_ > *ρf* + *g* does not hold.

Figure 4*c* shows the outcomes when *x*_*n*_ − *g* < *ρf* < *x*_1_; we find the bistability of the all cooperation and the first group defection equilibrium. When *x*_*n*_ − *g* < *x*_1_ < *x*_*j*−1_ < *ρf* < *x*_*j*_ < *x*_*n*−1_ there exists the bistability of the cooperation–defection mixed equilibrium and the all cooperation equilibrium. When *x*_*n*_ − *g* < *x*_*n*−1_ < *ρf* there only exists the all cooperation equilibrium. The last group defection equilibrium is not locally stable.

Figure 4*d* shows that first role sanction system can never create all cooperation, even with very high punishments *f* and along all the initial cooperation frequency.

In sum, the defector sanction system works as sanction and promotes the evolution of cooperation when *x*_1_ < *x*_2_ < · · · < *x*_*n*_ and *ρf* is large enough. The first role sanction system does not work and it is equivalent to the baseline system. The numerical analyses indicate that *c_j_* becomes almost zero so that this parameter does not influence the simulation outcomes when *j* is the first defector group (figure 4).

### 3.3. Other cases

**Figure 6.**
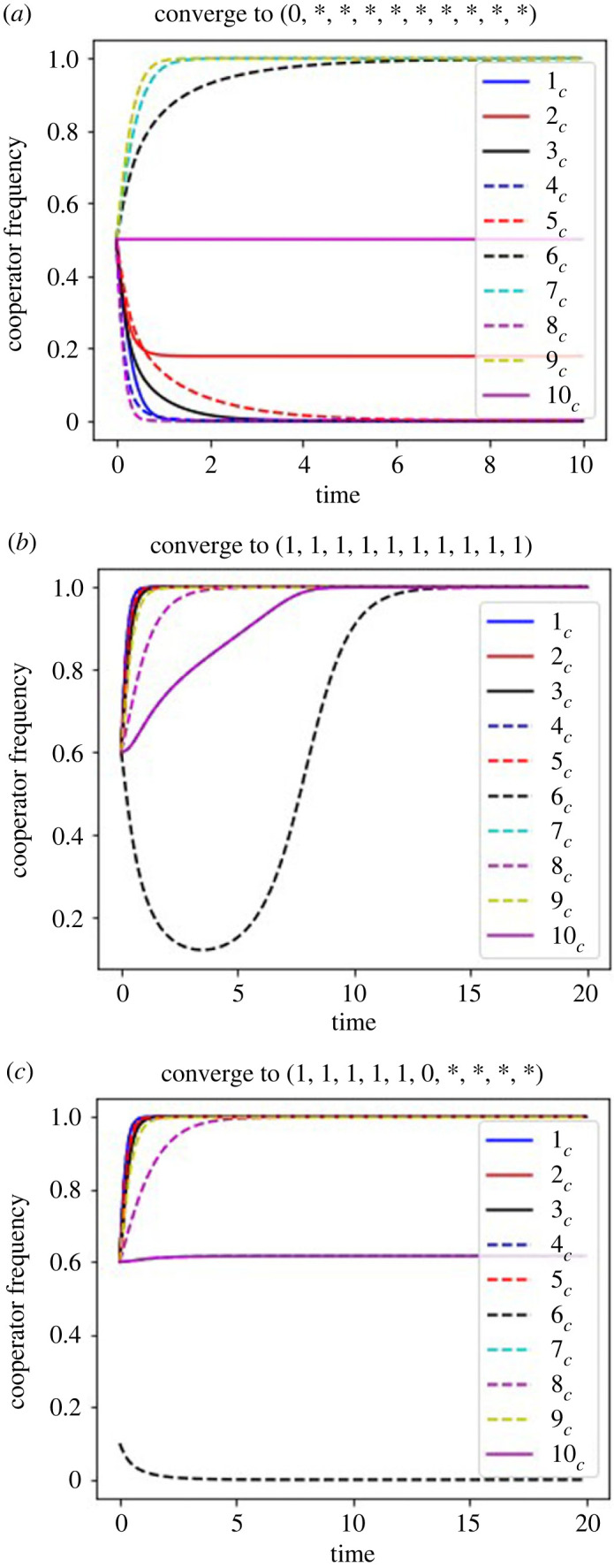
Time evolution with the costs of the cooperation given uniform randomly in the defector sanction system. In (*a*), *n* = 10, *g* = 3, *ρf* = 9, [*x*_1_, *x*_2_, …, *x*_10_] = [14, 20, 17, 20, 13, 6, 5, 19, 4, 9]. In (*b*,*c*), *n* = 10, *g* = 3, *ρf* = 10, [*x*_1_, *x*_2_, …, *x*_10_] = [4, 5, 6, 4, 5, 12, 10, 9, 7, 10]. The initial condition for (*a*) is *i_c_* = 0.5, and (*b*) is *i*_*c*_ = 0.6 for all the groups, and that for (*c*) is 6_*c*_ = 0.1 and *i*_*c*_ = 0.6 for other groups.

We consider that the costs of the cooperation are given uniform randomly, do the numerical simulations in a parameter set, and then see if tables 6–9 can predict the dynamics. Figure 6*a* shows that the dynamics approximately converges to [1_*c*_, 2_*c*_, 3_*c*_, 4_*c*_, 5_*c*_, 6_*c*_, 7_*c*_, 8_*c*_, 9_*c*_, 10_*c*_] = [0, 0.18, 0, 0, 0, 1, 1, 0, 1, 0.5] when [*x*_1_, *x*_2_, …, *x*_10_] = [14, 20, 17, 20, 13, 6, 5, 19, 4, 9] in the defector sanction system when *g* = 3 and *ρf* = 9. This convergence point seems to be a cooperation–defection mixed equilibrium. However, as *ρf* < *x*_1_, and *c*_1_ converges to zero in the simulation, table 6 predicts that it is the first group defection equilibrium. The simulation outcomes also show that, as 1_*c*_ converges to 0, it is the first group defection equilibrium. If we had only done the numerical analysis by computer simulations, we would have regarded this convergent point as a cooperation–defection mixed equilibrium. Therefore, the theoretical proofs help us understand the dynamics correctly.

When [*x*_1_, *x*_2_, …, *x*_10_] is randomly assigned to [4, 5, 6, 4, 5, 12, 10, 9, 7, 10], we observe both the all cooperation equilibrium and the cooperation–defection mixed equilibrium are locally stable with different initial conditions. Here, *n* = 10, *g* = 3, *ρf* = 10. We set the initial condition for figure 6*b*, *i*_*c*_ = 0.6 for all the groups, hence the dynamics converges to the all cooperation equilibrium. Table 6 also predicts the all cooperation equilibrium is locally stable here as $\max{\left\{x_{i}\right\}}_{i=1}^{10}=11<\rho f+g=13$. If we set the initial condition as 6_*c*_ = 0.1 and *i*_*c*_ = 0.6 (*i* ≠ 6), the dynamics converge to the cooperation–defection mixed equilibrium where players in the group 6 are changed to defectors (figure 6*c*). This can be predicted by table 6 in which $\max{\left\{x_{i}\right\}}_{i=1}^{5}=6<\rho f=10<x_{6}-c_{6}g=10.86$, where *c*_6_ converges to 0.379 in the simulation (table 6).

## 4. Discussion and conclusion

We took a system of linear division of labour where there are *n* roles (*n* ≥ 2). If a role gets subjected to defection by its defector, the labour stops there, and the players associated with the later roles do not get a chance to play the roles. Each player in each group gets subjected to the same loss once a player defects. We analyse three systems: the baseline system and the two sanction systems, namely the defector sanction system and the first role sanction system, to see their effect on the evolution of cooperation. After applying the replicator equation of asymmetric game, we find four equilibria, (i) where all the players in the first group are defectors, (ii) where all the players in all the groups are cooperators, (iii) where the players in the earlier groups are all cooperators and the players in the later groups except the first and the terminal group are defectors, which is called the cooperation–defection mixed equilibrium, and (iv) the last group defection equilibrium, where all the players in the last group are defectors, and all players in other groups are cooperators.

Our findings are as follows: the benefit given by a cooperator in an upstream group to a player in a downstream group does not influence the evolutionary dynamics, but the cost of cooperation does. We compared two sanction systems, the defector sanction system and the first role sanction system, with the baseline system. Then, we found that the defector sanction system promotes the evolution of cooperation unless the probability of finding a defector is very low. However, when it is too hard to monitor and detect a defector, the defector sanction system does not work as sanction any more. The first role sanction system promotes cooperation when the cost of cooperation decreases in downstream groups. Otherwise, the first role sanction system is equivalent to the baseline system; it does not work as sanction. The other important point is that, in addition to the all cooperation and the first group defection equilibria, the cooperation–defection mixed equilibrium and the last group defection equilibrium can be locally stable when the cost of cooperation increases with higher *i* in the defector sanction system.

Even though our results can be applied to any *n* if *n* ≥ 2, the results in *n* = 2 are different from those in *n* ≥ 3. The crucial difference is that there are three equilibrium points in *n* = 2: [1_*c*_, 2_*c*_] = [0, ∗], [1, 0], [1, 1]. When the costs of the cooperation decrease downstream, the local stability condition in *n* = 2 is the same as in *n* ≥ 3. When the costs of the cooperation increase downstream in the defector sanction system, in *n* = 2, [1,0] is locally stable if *x*_1_ < *ρf* and *x*_2_ > *ρf* + *g*. [1,1] is locally stable if *ρf* + *g* > *x*_2_, and [0,*] is locally stable if *ρf* < *x*_1_. There is a bistable region in *n* = 2; if *ρf* < *x*_1_ < *x*_2_ < *ρf* + *g* holds, [0,∗] and [1,1] are locally stable, while [1, 1, …, 1] and [0, ∗, …, ∗] are bistable in *n* > 2, when *x*_*n*_ − *g* < *ρf* < *x*_1_ (figure 4*c*). However, when *n* is larger, it becomes harder to have a bistable region in which [1, 1, …, 1] and [0, ∗, …, ∗] are locally stable, because *x*_*n*_ − *g* < *ρf* < *x*_1_ < · · · < *x*_*n*_ should be held under the assumption that *g* < *x*_*i*_.

Our study might remind us of Boyd & Richerson [59]. Because players are in a unidirectional cycle network, two neighbours play the PD game in order and it goes on repeatedly. The two strategies are as follows: (i) upstream tit for tat (UTFT): where if the upstream player cooperates/defects with the focus player, then he cooperates/defects with the downstream player and (ii) downstream tit for tat (DTFT): where the focus player cooperates/defects with the downstream player if the downstream player cooperated/defected with his own downstream player, in the previous cycle. Boyd & Richerson [59] found that DTFT evolved more than UTFT. Structurally this study might look similar to ours. However, there are some critical differences between the two. One difference is the research purpose; Boyd & Richerson [59] investigated the evolution of indirect reciprocity. The network structure of Boyd & Richerson [59] is a repeated cycle, and players observe the payoff of all players in a cycle and imitate the strategy of a player with a higher payoff. Basically, in our study, UTFT is not possible as, if the player in the upstream group defects, the game stops there and the players from the focus group do not get to choose their strategy. DTFT is not possible as the strategy of a player is premeditated and does not depend on downstream groups’ player’s choosing. However, Boyd & Richerson [59] give us a hint to develop the study of the division of labour. In our future work, we will apply some of their assumptions into our framework, and modify and develop our study.

**Figure 7.**
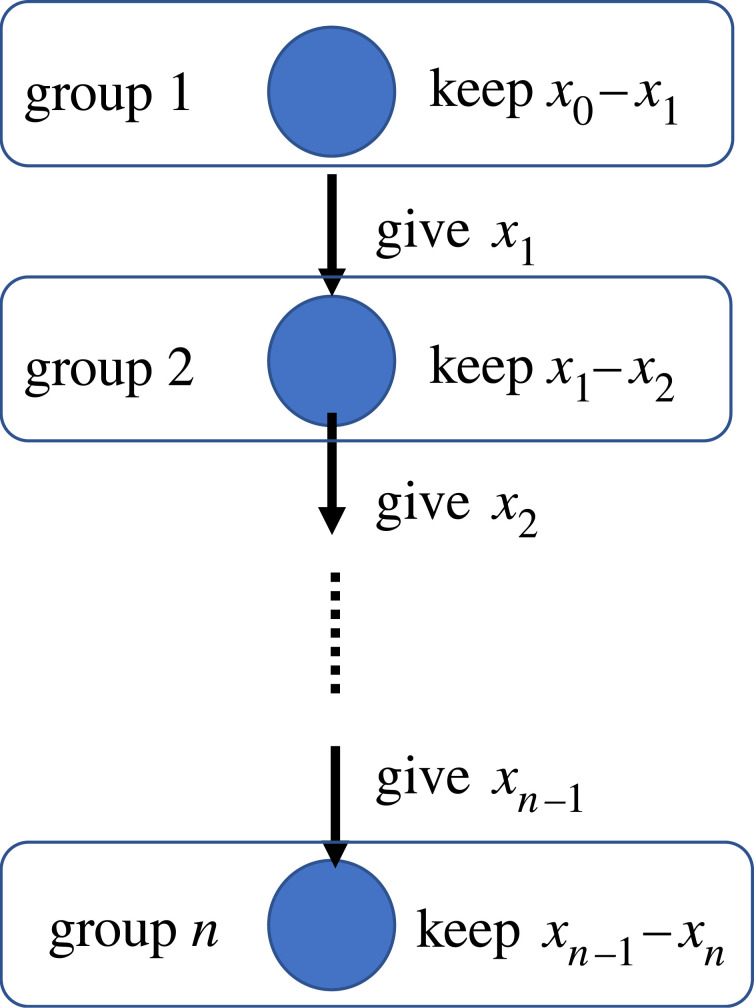
Allocation of the benefit among the players as an application of our model to government planning and spending. The net benefit of a player in group *i* is equal to the amount that the player keeps. This figure shows when all players in all groups are cooperators.

We consider the special case that *b*_*i*_ = *x*_*i*−1_, which means the benefit given by a cooperator in group *i* − 1 is the same as the cost of cooperation paid by the cooperator; a player in the first group gives 10 000 yen to a player in the second group. 10 000 yen is the cost of the player in the group 1 and the benefit to the player in the group 2. As *b*_*i*_ − *x*_*i*_ = *x*_*i*−1_ − *x*_*i*_ should be positive, *x*_*i*_ decreases as the *i* increases. Therefore, the result of the analyses corresponds to table 8. The total sum of the net benefit of all cooperators in all roles is (*x*_0_ − *x*_1_) + (*x*_1_ − *x*_2_) + · · · + (*x*_*n*−1_ − *x*_*n*_) = *x*_0_ − *x*_*n*_; therefore, the assumption that this situation can be interpreted as that each player in each role decides how much the upstream player keeps and distributes to the downstream players (figure 7). For example, a cooperator in group 1 keeps *x*_0_ − *x*_1_ and gives *x*_1_ to a player in group 2. If the player in the group 2 is a cooperator, the cooperator keeps *x*_1_ − *x*_2_ and gives *x*_2_ to a player in the group 3. This continues before a player chooses defection. This situation has some implications not only for the division of labour but also for government planning and spending or subcontract because the model can be assimilated with the flow of government spending or subcontract (see figure 7). For government planning and spending, cooperation in the division of labour is required [60]. Government of all forms performs a very significant role in running modern country states. The roles of it are as elaborated as they can get and a single individual cannot perform all roles. Therefore, all the related tasks are divided among many ministries and the ministries also divide the tasks among their workers. For a certain work to be done at the root level, the fund should go through multiple agents as well as be planned by multiple agents. Therefore, cooperation of all the roles is key for success in that particular task. For example, if a governmental head wants to spend some money at the root level, he/she allocates the money to his/her subordinate, who also allocates a part of the money to his/her subordinate, and it goes to many levels of subordinates until reaching the goal. Every cooperator in the group *i* here gains the net benefit *x*_*i*−1_ − *x*_*i*_ from cooperation (see figure 7). And if someone stops or does not cooperate because of corruption or other reasons, the money does not reach the goal and therefore the task fails. As a result, all players additionally get damage, −*g*. In return, the defector, of course, gains the money which was given to him but to every agent as a part of the government comes a bad reputation for the defection which can be set as −*g*. By creating cooperation among all the roles, we make sure that the players in the first role engaging in government spending do not choose defection. The reason is as follows; our results suggest that all cooperation equilibrium or first group defection equilibrium can be locally stable but the cooperation–defection mixed equilibrium is not stable in table 8. This means if a player in the first group is a cooperator, players in all other groups can be cooperators.

We did not comment particularly on the impact of the net benefit *b*_*i*_ − *x*_*i*_ or the distribution of the benefit for the cooperators in the system because the benefit does not influence the dynamics (see tables 6–9). Nowak [8] shows that the cooperation can evolve in the network-structured population, where each node has *k* regular links and *b* is the benefit from a cooperator and *c* is a cost of cooperation, in *b*/*c* > *k*. However, in our work, the benefit from cooperation is cancelled out, and then we cannot summarize our result using the benefit *b*. In this point, our work shed a new light on the evolution of cooperation in the networks.

This work only focused on one case of the division of labour. There are other types of division of labour. Here we assumed that each player in each group obtained the payoff after the player plays the game with the player in the downstream group. While, in the other type of division of labour, each player can get the benefit after all tasks in the division of labour are completed. In our future work, we will investigate different outcomes we will obtain by analysing the various cases of the division of labour by the replicator equations of asymmetric games. In addition, we will consider other types of sanction: for example, mistakenly regarding cooperators as defectors and sanctioning them.

## Data Availability

Data available from the Dryad Digital Repository: https://doi.org/10.5061/dryad.k3j9kd5b2 [61].

## Appendix A

**A.1. Proof of the equilibrium**

Assumption, *x*_*i*_ > 0, *x*_*i*_ ≠ *g* for all *i*’s. From the model setting we also get when, *i*_*c*_ = 0, *j*_*c*_ = *, if, *j* > *i*. In an equilibrium, equations (2.1)–(2.3) are zero.

We know, d*n*_*c*_/d*t* = 0 ⇒ *n*_*c*_ = 0, or *n*_*c*_ = 1, or *c*_*n*_ = 0 · · · (*α*)

When *n*_*c*_ = 0 in (*α*), *c*_1_ = 0. Therefore, 1_*c*_ = 0, or 1 (as, *x*_1_ ≠ 0).

When *n*_*c*_ = 1 in (*α*), (*n* − 1)_*c*_(1 − (*n* − 1)_*c*_){*c*_*n*−1_*g* − *x*_*n*−1_(1 − *d*_(*n*−1)*b*_)} = 0. (*n* − 1)_*c*_ ≠ 0 (as *n*_*c*_ = 1, and all players in the later group cannot be cooperators if all players in the former group are defectors in our model). {*c*_*n*−1_*g* − *x*_*n*−1_(1 − *d*_(*n*−1)*b*_)} = 0 ⇒ *g* = *x*_*n*−1_ (as *c*_*n*−1_ = 1 − *d*_(*n*−1)*b*_). However, it is a contradiction. Therefore, only (*n* − 1)_*c*_ = 1 is possible. Following the same logic upstream, we get 1_*c*_ = 1.

When *c*_*n*_ = 0 in (*α*), there exists *k* = {1, …, *n* − 1} such that *k*_*c*_ = 0. When *k* ≠ 1, *c*_1_ = 0. Therefore, 1_*c*_ = 0, or 1.

Therefore, all the cases in (*α*) lead to 1_*c*_ = 0, or 1 when in equilibrium. When 1_*c*_ = 0, the equilibrium is the first group defection equilibrium.

When 1_*c*_ = 1, 1 − *d*_2*b*_ = 1 · · · (*β*)

Therefore, d2_*c*_/d*t* = 2_*c*_(1 − 2_*c*_){*c*_2_*g* − *x*_2_} = 0. By repeating the process from (*α*) we get 2_*c*_ = 0, or 1. When 2_*c*_ = 0 the equilibrium is a cooperation–defection mixed equilibrium. When 2_*c*_ = 1, the process shall be repeated from (*β*) to get the equation for 3_*c*_. By repeating this we finally get when [1_*c*_, …, (*n* − 1)_*c*_] = [1, …, 1], *n*_*c*_ = 0, or 1. When *n*_*c*_ = 0 it is the last group defection equilibrium, and when *n*_*c*_ = 1 it is the all cooperation equilibrium.

## Appendix B

We analysed the local stability of the equilibria in each of the three systems for the general model.

For the baseline system, all eigenvalues of the Jacobian matrix for the first group defection equilibrium are *c*_1_*g* − *x*_1_ and 0. Therefore, the first group defection equilibrium is stable, as *c*_1_*g* − *x*_1_ < 0. The cooperation–defection mixed equilibrium and the last group defecting equilibrium are unstable, as the eigenvalue *x*_1_ is positive and it is always an eigenvalue for both of these as the first defector *j* ≥ 2. All eigenvalues of the Jacobian matrix for the all cooperation equilibrium are *x*_*i*_ − *g*, (1 ≤ *i* ≤ *n*). Therefore, the all cooperation equilibrium is stable in the baseline system if max{*x*_*i*_}_1≤*i*≤*n*_ < *g*, for all the 1 ≤ *i* ≤ *n*. We consider the PD situation in the baseline model, therefore, *x*_*i*_ > *g*. This indicates that all the cooperation equilibrium is unstable in the baseline.

For the defector sanction system, all eigenvalues of the Jacobian matrix for the first group defection equilibrium are *c*_1_*g* + *ρf* − *x*_1_ and 0. Therefore, the first group defection equilibrium is stable in the defector sanction system if *c*_1_*g* + *ρf* < *x*_1_. The cooperation–defection mixed equilibrium in the defector sanction system is stable if max{*x*_*i*_}_*i*<*j*_ < *ρf* and *x*_*j*_ − *c_j_g* > *ρf* when *j* is the first defector. The last group defection equilibrium is stable when $\max{\left\{x_{i}\right\}}_{i=1}^{n-1}<\rho f$ and *x*_*n*_ > *ρf* + *g*. All eigenvalues of the Jacobian matrix for the all cooperation equilibrium are *x*_*i*_ − *g* − *ρf* (1 ≤ *i* ≤ *n*). Therefore, the all cooperation equilibrium is stable in the defector sanction system if *g* + *ρf* > max{*x*_*i*_}_1≤*i*≤*n*_.

For the first role sanction system, all eigenvalues of the Jacobian matrix for the first group defection equilibrium are *c*_1_(*g* + *f*) − *x*_1_ and 0. Therefore, the first group defection equilibrium is conditionally stable. For the equilibrium to be a mixed equilibrium, *x*_1_ is always an eigenvalue when the first defector *j* ≥ 2. As a result, the cooperation–defection mixed equilibrium and the last group defection equilibrium are unstable, as the eigenvalue *x*_1_ is positive. All eigenvalues of the Jacobian matrix for the all cooperation equilibrium are *x*_*i*_ − *g* (2 ≤ *i* ≤ *n*) and *x*_1_ − *g* − *f*. Therefore, the all cooperation equilibrium is stable in the first role sanction system if *g* + *f* > *x*_1_ and *g* > max{*x*_*i*_}_2≤*i*≤*n*_.
